# Variations in the Path From Bench to Bedside in Translational Research on Caregiving

**DOI:** 10.1093/geroni/igz042

**Published:** 2019-11-14

**Authors:** J Jill Suitor

**Affiliations:** Department of Sociology, Purdue University, West Lafayette, Indiana

Caregiving has been a central topic within the field of aging for more than four decades. Across this period, research has been influenced by changing demographics, including an unprecedented growth in the proportion of the population over the age of 65 (U.S. Census Bureau Population Division, 2019) and a commensurate increase in the number of individuals, particularly family members, providing care to older adults (National Alliance for Caregiving and AARP, 2015). In fact, more than 150,000 articles, books, and book chapters on the topic have been published since 1970, with the number of publications increasing each year (Google Scholar). As the demands on family members to provide care to their elders, and the physical, psychological, and financial costs of such demands have become widely acknowledged (Adelman, Tmanova, Delgado, Dion & Lachs, 2014; Caputo, Pavalko & Hardy, 2016), there have been increasing calls to develop evidence-based interventions to improve the lives of caregivers and their care recipients, as well as to disseminate and implement existing interventions (Gitlin, Marx, Stanley, & Hodgson, 2015; Pillemer & Gilligan, 2018; Qualls, 2016; Wethington & Burgio, 2015).

Our goal in this special issue was to respond to these calls by bringing together a set of papers on translational research on caregiving that would span a broad range of methods and topics, both introducing new approaches and extending approaches that are well-established. In defining translational research, we drew from that offered by Pillemer and Gilligan:

Systematic translation of research findings from gerontology into the development of innovative interventions that ultimately improve real-world practices and from interventions and practices back to basic research (2018:2).

Our call for submissions emphasized our interest in studies that would contribute to the translation of scientific discoveries into methods, interventions, and treatments that would improve the everyday lived experiences of caregivers and care recipients. We received 64 abstracts in response to our call, a number far greater than we could include. From these, we selected 11 for development into the full articles that comprise the special issue.

We believe that the special issue meets the benchmark we set for translational approaches by including articles on new interventions that provide opportunities to improve the lives of care recipients and their caregivers, expand existing successful interventions to broader contexts, highlight understudied populations of caregivers, and offer both quantitative and qualitative approaches to the study of caregiving. Some of the articles we have included also took us in directions beyond our original vision of the issue by providing new and unique windows on the experiences of caregivers and care recipients, and exploring older adults’ agency regarding their preferences for care and residential setting, both of which have implications for current and future caregivers.

The articles by Bass and colleagues (2019) and Cho, Luk-Jones, Smith, and Stevens (2019) provide excellent examples of implementing interventions, known to work in highly controlled settings, to broader contexts in which they can improve the lives of a larger segment of the population of caregivers and care recipients. Bass and colleagues implemented and evaluated the evidence-based “Partners in Dementia Care,” a personalized coaching program for caregivers in a real-world setting through partnerships between a Department of Veterans Affairs Medical Center and both a chapter of the Alzheimer’s Association and an Area Agency on Aging. The evaluation demonstrated improved outcomes for both caregivers and care recipients when integrated into the regular regime of services offered that were consistent with those found in prior randomized trials. Similarly, Cho and colleagues (2019) evaluated the implementation of REACH-TX, a modified version of the REACH-II (Resources for Enhancing Alzheimer’s Caregiver Health), an intervention designed to provide caregivers with evidence-based skills training and support which has previously been tested in health care settings. The purpose of Cho and colleagues’ work was to evaluate the effectiveness of the REACH-TX in a community setting. Implementation of the intervention was facilitated by partnering with the staff at a state chapter of the Alzheimer’s Association who both recruited participants and delivered the intervention. Consistent with Bass and colleagues, Cho and colleagues found improvements in caregivers’ outcomes that were similar to those found in randomized controlled trials. Taken together, these two studies demonstrated that interventions found to be efficacious in randomized control trials could be effective in “real-world” settings through partnerships with institutions and agencies in the community, increasing the number of caregivers and care recipients whose quality of life can be improved.

Three articles included in the special issue highlight how markedly different interventions can be implemented to improve the lives of caregivers and care recipients (Leszko, 2019; Muramatsu & Yin, 2019; Williams et al., 2019). Williams and colleagues (2019) present results from a technology-based intervention (FamTechCare) in which dementia caregivers in the experiential group submitted weekly videos of care situations over a 3-month period. The videos were then reviewed collectively by a team of dementia experts, after which caregivers received individualized interventions in a weekly phone call with one of the dementia experts. In the control group, caregivers participated in weekly telephone calls with a member of the team of dementia experts, retrospectively describing their recent challenging experiences and discussing interventions to address these challenges. Despite what may appear to be small differences in the delivery of support to in these two conditions, caregivers in the experimental group had much greater reductions in depression and gains in confidence than did those in the “attention” control group. The findings suggest that mode of participation and delivery may play an important role in the effectiveness of technological support interventions to dementia caregivers.

Muramatsu and Yin (2019) demonstrate the importance of mode of delivery of interventions in the unique context of a physical activity intervention. Family and nonfamily health care aides received the same half-day training on motivational enhancement and gentle physical activity, which they delivered to their care recipients across a 4-month period. The findings revealed that although care recipients in both groups showed improvements in physical functioning and exercise-related social support, gains were greater when the intervention was delivered by nonfamily than family health care aides, thus highlighting the salience of the mode of delivery.

Finally, in this group of new interventions, Leszko (2019) studied the impact of providing psychoeducational and financial interventions to dementia caregivers in Poland, a cultural context that has received little attention in the literature. Leszko begins by demonstrating that spousal caregivers who received both psychoeducational training and a stipend from the local government reported declines in depression and caregiver burden over a 6-month period, whereas caregivers who only completed interviews and questionnaires did not. By employing a mixed-method design, she was also able to shed light on the important role that the financial dimension of the intervention played in caregivers’ day-to-day lives and plans for the future,

Four of the articles in the special issue used innovative methods to study caregiver outcomes that provide valuable evidence that can be used in the development of caregiver interventions. Two of these studies, Rigby, Ashwill, Johnson, and Galvin (2019) and Chiriboga, Park, Gilbert, Molinari, and Barnes (2019), consider contextual factors affecting the experiences of dementia caregivers. Rigby and colleagues (2019) studied differences in the well-being of adult children and spouses caring for persons with dementia with Lewy Bodies, finding that although adult children spent less time with the person with dementia (PWD) than did spouses, they reported lower quality of life and more caregiver burden than did spouses. The findings from this study have important implications for the development of interventions designed to improve the lives of Lewy Body caregivers. Because Dementia with Lewy Bodies (DLB) is less common than Alzheimer’s Disease (AD), the need for effective interventions, particularly for adult children, have been given relatively little attention in the literature, despite the higher level of patient disruptive behaviors and greater caregiver stress associated with DLB. Thus, we believe that Rigby and colleagues’ study highlights an important gap in the development of interventions targeting dementia caregivers.

Chiriboga and colleagues’ (2019) investigation of PWD enrolled in dementia-specific adult day centers and their caregivers also addresses an important yet understudied contextual dimension of caregiving—the role of adult day centers for caregivers to PWD. Their central aims were identifying the average levels of impairment of the PWD at the time of enrollment in the center and burden of their caregivers, and how these markers varied by race and ethnicity. Although the average levels of impairment did not differ by race or ethnicity, Latinx caregivers reported the greatest burden, whereas Black caregivers, especially daughters, reported the least. These findings highlight several important points that should be considered in developing and evaluating caregiving interventions, including greater attention to exploring the role of adult day centers in dementia caregivers’ well-being, as well as how interventions may differ in effectiveness by race, ethnicity, and gender.

The articles by Beach, Kinnee, and Schulz (2019) and Roberto, McCann, Blieszner, and Savla (2019) are examples of innovative methodological approaches that provide new prisms through which to understand the factors that shape experiences of caregivers and inform the development of interventions to improve the quality of their lives. Beach and colleagues’ (2019) study brought together traditional survey and Geographic Information System techniques to explore the role of neighborhood characteristics in variations in the experiences and needs of family caregivers. Data were collected in telephone interviews with caregivers whose neighborhoods were classified on the basis of Environmental Justice Areas (EJAs) and Medically Underserved Areas (MUAs). Contrary to expectations, caregivers living in neighborhoods classified as both EJAs and MUAs were less likely to report depression and showed a trend of reporting more positive aspects of caregiving than those living in non EJA/MUA settings. Thus, Beach and colleagues’ findings demonstrate the salience of questioning assumptions that are often made about the experiences, and potentially the needs, of caregivers in under-resourced neighborhoods when developing interventions to assist their residents.

Roberto and colleagues (2019) bring a new perspective to understanding the needs and experiences of family caregivers by taking an innovative approach to applying a classic method in social science. Roberto and colleagues conducted in-depth interviews four times over a decade with 10 women caring for older spouses or parents, beginning after the PWD had been diagnosed with mild cognitive impairment. Using “grit” to conceptualize the women’s commitment to their roles as caregivers over an extended and increasingly challenging period, Roberto and colleagues described the pathways by which they managed their identities and role engagement across time. Their research highlights the salutary effects of consciously taking charge and making decisions for themselves and the PWD for whom they cared, including pursuing employment and leisure activities despite their caregiving demands. The findings from this investigation confirm the importance of applying longitudinal qualitative methods to identify aspects of role management of dementia caregivers that may be missed using cross-sectional and quantitative approaches, yet may be essential to consider when developing interventions to improve well-being across the caregiving career.

Finally, two of the articles in this special issue focus on older adults’ agency regarding their current and future preferences for care and day-to-day living. Torres and Cao (2019) use qualitative data from several years of ethnographic field work to explore older adults’ use of and preferences for spending time in “third places” (Oldenburg & Brissett, 1982), a term used to refer to places outside of home (first places) and work (second places) where people spend time. Torres and Cao’s work highlights the increasing salience of informal age-integrated third places as individuals age, yet may not want to spend their time outside of their homes in age-based, age-segregated settings such as senior centers or formal health care settings. Understanding the preferences of seniors while they are still living in the community and do not yet need formal or informal care may play an essential role in designing interventions for family members who later become caregivers to elders for whom maintaining their lives in age-integrated settings is highly salient.

Dassel, Utz, Supiano, Bybee, and Iacob (2019) offer a new dementia-focused tool for end-of-life planning. They developed the LEAD guide (Life-Planning in Early Alzheimer’s and Dementia) through multiple phases in which they took a mixed-method approach involving, in turn, focus groups, content evaluation by a panel of experts, completion of the tool by both healthy older adults and those with early-stage ADRD, followed by focus groups with caregivers, older adults with early-stage ADRD, and healthy older adults to provide guidance on the use of the LEAD in informal and clinical settings. Although a large and growing literature exists on advance care planning, there has been little attention directed toward developing an ACP tool for use with adults at high risk of developing ADRD or who have already begun to show symptoms. Thus, the availability of the LEAD guide is likely to have far-reaching implications for families, clinicians, and researchers developing interventions to meet the needs of caregivers and care recipients as they embark on this difficult journey.

Collectively, this special issue presents a set of articles on translational caregiving that showcase innovative interventions that can be applied today in real-world settings, highlight sociodemographic and contextual factors that are important to take into consideration when designing and implementing caregiving interventions, and present valuable information about the experiences of caregivers and care recipients that can provide the basis for the development of new interventions. The wide range of questions addressed and methodologies employed in these articles, taken together, speak to the full caregiving career, from the point immediately before individuals experience this transition to late-stage dementia and planning for the end of life. We hope that these articles will spur translational research in new directions that further improve the lives of caregivers and the older adults for whom they care.
